# Pathological validation and prognostic potential of quantitative MRI in the characterization of pancreas cancer: preliminary experience

**DOI:** 10.1002/1878-0261.12688

**Published:** 2020-06-23

**Authors:** Remy Klaassen, Anne Steins, Oliver J. Gurney‐Champion, Maarten F. Bijlsma, Geertjan van Tienhoven, Marc R. W. Engelbrecht, Casper H. J. van Eijck, Mustafa Suker, Johanna W. Wilmink, Marc G. Besselink, Olivier R. Busch, Onno J. de Boer, Marc J. van de Vijver, Gerrit K. J. Hooijer, Joanne Verheij, Jaap Stoker, Aart J. Nederveen, Hanneke W. M. van Laarhoven

**Affiliations:** ^1^ Department of Medical Oncology Cancer Center Amsterdam Amsterdam UMC University of Amsterdam The Netherlands; ^2^ Laboratory for Experimental Oncology and Radiobiology Center for Experimental and Molecular Medicine Cancer Center Amsterdam Amsterdam UMC University of Amsterdam The Netherlands; ^3^ Department of Radiology & Nuclear Medicine Cancer Center Amsterdam Amsterdam UMC University of Amsterdam The Netherlands; ^4^ Department of Radiation Oncology Cancer Center Amsterdam Amsterdam UMC University of Amsterdam The Netherlands; ^5^ Oncode Institute Amsterdam The Netherlands; ^6^ Department of Surgery Erasmus Medical Center Rotterdam The Netherlands; ^7^ Department of Surgery Cancer Center Amsterdam Amsterdam UMC University of Amsterdam The Netherlands; ^8^ Department of Pathology Cancer Center Amsterdam Amsterdam UMC University of Amsterdam The Netherlands

**Keywords:** carcinoma, pancreatic ductal, magnetic resonance imaging, diffusion magnetic resonance imaging, histological techniques, prognosis

## Abstract

Patient stratification based on biological variation in pancreatic ductal adenocarcinoma (PDAC) subtypes could help to improve clinical outcome. However, noninvasive assessment of the entire tumor microenvironment remains challenging. In this study, we investigate the biological basis of dynamic contrast‐enhanced (DCE), intravoxel incoherent motion (IVIM), and R2*‐derived magnetic resonance imaging (MRI) parameters for the noninvasive characterization of the PDAC tumor microenvironment and evaluate their prognostic potential in PDAC patients. Patients diagnosed with treatment‐naïve resectable PDAC underwent MRI. After resection, a whole‐mount tumor slice was analyzed for collagen fraction, vessel density, and hypoxia and matched to the MRI parameter maps. MRI parameters were correlated to immunohistochemistry‐derived tissue characteristics and evaluated for prognostic potential. Thirty patients were included of whom 21 underwent resection with whole‐mount histology available in 15 patients. DCE *K* ^trans^ and *v*_e_, ADC, and IVIM *D* correlated with collagen fraction. DCE *k*_ep_ and IVIM *f* correlated with vessel density and R2* with tissue hypoxia. Based on MRI, two main PDAC phenotypes could be distinguished; a stroma‐high phenotype demonstrating high vessel density and high collagen fraction and a stroma‐low phenotype demonstrating low vessel density and low collagen fraction. Patients with the stroma‐high phenotype (high *k*_ep_ and high IVIM *D*, *n* = 8) showed longer overall survival (not reached vs. 14 months, *P* = 0.001, HR = 9.1, *P* = 0.004) and disease‐free survival (not reached vs. 2 months, *P* < 0.001, HR 9.3, *P* = 0.003) compared to the other patients (*n* = 22). Median follow‐up was 41 (95% CI: 36–46) months. MRI was able to accurately characterize tumor collagen fraction, vessel density, and hypoxia in PDAC. Based on imaging parameters, a subgroup of patients with significantly better prognosis could be identified. These first results indicate that stratification‐based MRI‐derived biomarkers could help to tailor treatment and improve clinical outcome and warrant further research.

## Graphical abstract

Patient stratification based on biological variation in pancreatic ductal adenocarcinoma subtypes could help to improve clinical outcome. In this study, quantitative magnetic resonance imaging (MRI) parameters were directly correlated to immunohistochemistry‐derived tissue characteristics. Furthermore, based on MRI parameters a subgroup of patients with significantly better prognosis could be identified, indicating that patient stratification based on MRI parameters is feasible.

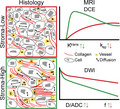

**Abbreviations**
ADC: apparent diffusion coefficient
CT: computed tomography
D*: pseudodiffusion coefficient
*D*: diffusion coefficient
DAB: 3,3′‐diaminobenzidine
DCE: dynamic contrast‐enhanced
DFS: disease‐free survival
DWI: diffusion‐weighted imaging
*f*: perfusion fraction
HIER: heat‐induced epitope retrieval
HIF‐1α: hypoxia‐inducible factor 1‐alpha
IVIM: intravoxel incoherent motion
*k*_ep_: rate constant
*K*^trans^: transfer constant
MRI: magnetic resonance imaging
OS: overall survival
PDAC: pancreatic ductal adenocarcinoma
PSR: Picrosirius Red
ROI: region of interest
*v*_e_: extracellular extravascular space
*v*_p_: blood plasma volume
VWF: von Willebrand factor

## 1 Introduction

The severe desmoplastic reaction often present in pancreatic ductal adenocarcinoma (PDAC) has been associated with dismal prognosis and therapy resistance (Özdemir *et al.*, 2014). This desmoplastic reaction involves extensive fibrosis, severe immune infiltration, and hypovascularization (Feig *et al.*, 2012). As a result of increased interstitial pressure and reduced vascularization, pancreatic tumors often present with high levels of hypoxia (Koong *et al.*, 2000). Variation in these three biological characteristics of PDAC – desmoplasia, hypovascularization, and hypoxia – have been related to differences in treatment outcome (Bailey *et al.*, 2016; Puleo *et al.*, 2018).

Patient stratification based on this biological variation could help to tailor treatment and improve clinical outcome. However, characterization of the PDAC microenvironment in patients remains difficult, since (endoscopic) biopsies often yield too little tissue for full characterization and are prone to spatial sampling variation.

Quantitative magnetic resonance imaging (MRI), such as dynamic contrast‐enhanced (DCE), diffusion‐weighted imaging (DWI), and T2*‐weighted MRI, potentially enables noninvasive characterization of desmoplasia, hypovascularization, and hypoxia of the entire tumor (Gurney‐Champion *et al.*, 2018; Klaassen *et al.*, 2018a, 2018b,2018a, 2018b). In DCE MRI, imaging is performed repeatedly after contrast injection and quantified by fitting a multicompartment model to the tissue contrast uptake curve. DWI uses gradients placed prior to the signal readout to sensitize the MRI signal to the diffusivity of water molecules. Cellular structures hamper this water diffusivity, enabling DWI to characterize the tissue using a mono‐exponential function of the DWI signal decay. The intravoxel incoherent motion (IVIM) model (Le Bihan *et al.*, 1988) uses a bi‐exponential fit to also model the faster perfusion‐driven movement of water molecules in the capillaries, enabling a separate means of quantifying tissue perfusion. In R2* (the reciprocal of T2*‐relaxation time) MRI, the difference in magnetic permeability between oxy‐ and deoxyhemoglobin is exploited to determine tissue oxygenation. DCE (Bali *et al.*, 2011; Ma *et al.*, 2016) and IVIM (Klauss *et al.*, 2015; Lemke *et al.*, 2009) have demonstrated potential in characterizing PDAC lesions, DWI has shown prognostic relevance in PDAC patients (Heid *et al.*, 2016), and studies in other cancer types have shown the relation between hypoxia and R2* (Hoskin *et al.*, 2007). However, implementation of imaging biomarkers in the clinical workup of PDAC is not straightforward and still lacking. The exact interpretation of the MR parameters is greatly dependent on the underlying tissue conditions and used techniques. Direct correlation to histology and patient outcome is often lacking. In this study, we match surgery obtained pathology to the MRI in an unprecedented way to directly correlate the MRI parameters to histopathology‐derived tissue characteristics. Furthermore, we investigated whether these parameters can be used as noninvasive prognostic marker in patients with PDAC.

## 2 Materials and methods

### 2.1 Patients

For this prognostic study, patients were included at the Amsterdam UMC, location AMC, during November 2013 and November 2017. Inclusion criteria comprised computed tomography (CT)‐diagnosed high suspicion of resectable PDAC (Dutch Pancreatic Cancer Group criteria, Versteijne *et al.*, 2016), scheduled for surgical exploration, a minimal eGFR of 30 mL·min^−1^·1.73 m^−2^, and no contraindications to undergo MRI scanning. The study was approved by the institutional review board of the Academic Medical Center (METC2013_254, NCT01989000) and performed according to the standards set by the Declaration of Helsinki. All patients gave written informed consent before the start of the study. Patients did not receive any oncological treatment before MRI scans were performed. Complete clinical follow‐up was used until September 2018.

### 2.2 Magnetic resonance imaging and processing

Magnetic resonance imaging was performed on a 3T MR scanner (Ingenia, Philips, Best, the Netherlands) on which we obtained quantitative DCE, T2*, and DWI images. For anatomical verification, a multi‐echo spoiled gradient echo with three‐point Dixon reconstruction (mDIXON) sequence was performed 35 s after contrast injection. Relevant sequence parameters are summarized in Table 1.

**Table 1:** Summary of the relevant MRI sequence parameters for DCE, T1 mapping, DWI, T2*, and mDIXON acquisition. FOV, field of view; RL, right–left; AP, anterior–posterior; TR, repetition time; TE, echo time; TI, inversion time; FA, flip angle; Resp., respiratory.

	DCE	T1	DWI	T2*	mDIXON
Sequence type	Fast Field Echo	Look‐Locker	Echo Planar Imaging	Multi‐echo (8) Spoiled Gradient Echo	Multi‐echo (3) Spoiled Gradient Echo
FOV (RL × AP, mm^2^)	400 × 400	400 × 350	432 × 108	400 × 355	400 × 350
Acquisition matrix	160 × 160	132 × 116	144 × 34	176 × 154	236 × 208
Slice thickness/gap (mm)	2.5 (5.0 noninterpolated)	5.7 (11.4 noninterpolated)	3.7/0.3	2.3 (4.6 noninterpolated)	1.7
Slices	30	13	18	41	53
TR/TE1/ΔTE (ms)	3.2/2.0/–	3.5/1.6/–	> 2200/45/–	20/2.3/2.3	4.7/1.2/1.0
TI1/TI (ms)	–	19/85	–	–	–
FA (°)	20	8	90	12	25
SENSE (RL/AP)	3.6/1.5	3/1.3	1.3 AP	1.5FH/2AP	2/1.5
Scan time (total)	1.75 s (280 s)	24 s	~ 10 min	22 s	21 s
Resp. compensation	Postprocessing	1 breath hold	Resp. trigger (navigator)	1 breath hold	1 breath hold
	**DWI**				
*b*‐values (s·mm^−2^) and (directions/averages)	0 (15), 10 (9),20 (9), 30 (9), 40 (9), 50 (9), 75 (4), 100 (12), 150 (4), 250 (4), 400 (4), 600 (16)				
Diffusion times δ/Δ (ms)			10.1/22.6		

Image processing was performed using in‐house software written in matlab (R2015b; MathWorks, Natick, MA, USA), unless stated otherwise.

T2* and DCE data were obtained and processed as described in detail in our earlier performed repeatability study (Klaassen *et al.*, 2018b). A mono‐exponential function was used to model the signal intensity decay at different echo times to retrieve quantitative maps of T2* and R2* relaxation rate. A population‐based arterial input function was used derived from another set of pancreatic cancer patients using the same scan and injection protocol (Klaassen *et al.*, 2018b). The extended Tofts model was fitted for each voxel to retrieve parameter maps for the transfer constant (*K* ^trans^), rate constant (*k*_ep_ = *K* ^trans^/*v*_e_), extracellular extravascular space (*v*_e_), and blood plasma volume (*v*_p_). Voxels with unreliable fit results (*v*_e_ > 1.0) were discarded from further analysis.

Full details on DWI acquisition and data processing are described in our previous work, where the acquisition was optimized (Gurney‐Champion *et al.*, 2016) and used on a different set of PDAC patients (Gurney‐Champion *et al.*, 2018; Klaassen *et al.*, 2018a). Diffusion coefficient (*D*), perfusion fraction (*f*), and pseudodiffusion coefficient (D*) maps were obtained by fitting the IVIM model to the signal decay as function of b‐value using a least‐squares fit. Additionally, apparent diffusion coefficient (ADC) maps were obtained using a mono‐exponential fit to the signal decay as function of all acquired b‐values.

### 2.3 Histopathology processing and MRI matching

**Fig. 1:**
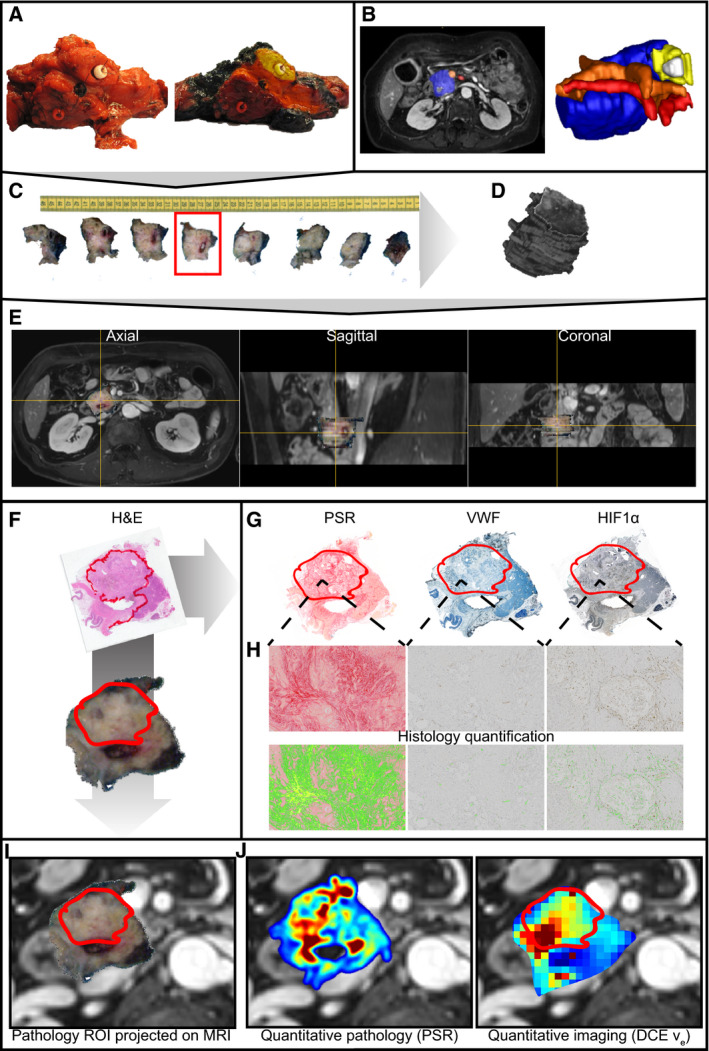
Graphical representation of the pathology to MRI matching procedure. (A) Anatomical structures are marked in the tissue specimen. (B) Anatomical structures are marked on the MRI. (C) The tissue specimen is sliced in axial‐oriented slices. (D) The tissue specimen is reconstructed in 3D MRI space by aligning the tissue slices. (E) The 3D reconstructed slices are projected onto the MRI and aligned to match anatomical structures visible on both MRI and pathology. (F) The whole‐mount slice is stained with H&E, and the tumor area is annotated by a pathologist. (G) The tumor ROI is copied to the immunohistochemistry of the whole‐mount slice. (H) The histology slices are quantified. (I) The pathology ROI is projected onto the matched MRI. (J) The ROI is propagated to the quantitative histology and MRI.

Directly after resection, colored beads were sutured to relevant anatomical structures (i.e., mesenteric vein and artery margins, bile duct, and pancreatic duct) and dissection planes of the resection specimen (Fig. 1A left) and marked by a pathologist using colored ink (Fig. 1A right). Independently, relevant anatomical structures were annotated on the MRI and reconstructed to form a 3D volume of the tumor area (Fig. 1B). After overnight fixation in 4% paraformaldehyde, the tissue was sliced in approximately 5‐mm‐thick axial‐oriented slices that were numbered and photographed from both sides. One complete tissue slice comprising evident tumor was selected for whole‐mount processing (Fig. 1C). Next, the photographed slices were arranged and automatically realigned to form a 3D volume of the pathology specimen using the image scale obtained from an on the photograph included ruler and an approximated slice thickness of 5 mm (imagej, stackreg, Thevenaz *et al.*, 1998) (Fig. 1D). Next, the colored landmarks in the 3D reconstructed pathology specimen were matched to the manual annotations in the MRI in 3d slicer (https://www.slicer.org; Fedorov *et al.*, 2012) (Fig. 1E). This way, each slice in the pathology specimen was matched to the MRI image slices assuming approximately the same axial orientation for the pathology and MRI slices as starting point. Furthermore, care was taken to find the best possible match between MRI and pathology for the whole‐mount processed slice.

### 2.4 Immunohistochemical staining and quantification

After fixation, tissue was dehydrated in a series of ethanol and embedded in paraffin. Four micrometer‐thick sections were cut on a Leica Polycut S Microtome (Reichert Inc., Depew, NY), and tissue sections were deparaffinized in xylene and rehydrated in a series of ethanol. Whole‐mount slides were histochemically stained with hematoxylin (Klinipath; VWR International, Radnor, PA, USA) and eosin (H&E) and stained for collagen with Picrosirius Red (PSR; Brunschwig, Basel, Switzerland). For immunohistochemical (IHC) staining, sections were incubated in 0.3% hydrogen peroxide in methanol for 10 min. For endothelial staining, heat‐induced epitope retrieval (HIER) was performed in 0.25% pepsin (Sigma, Saint Louis, MO, USA) in 0.01 m hydrochloric acid for 15 min at 37 °C. von Willebrand factor antibody (VWF, Agilent, Santa Clara, CA, USA) was diluted in normal antibody diluent (Klinipath, 1 : 2000), and sections were incubated at 4 °C overnight. For hypoxia staining, HIER was performed in Tris/EDTA buffer solution at pH 9.0 (Lab Vision PT Module, Thermo Scientific, Waltham, MA, USA) for 15 min at 98 °C. Hypoxia‐inducible factor 1‐alpha (HIF‐1α) antibody (Clone 54, BD Biosciences, Franklin Lakes, NJ, USA) was diluted in normal antibody diluent (1 : 100), and sections were incubated at 4 °C overnight. Subsequently, for all IHC stainings BrightVision+ post‐antibody block was applied on the sections for 15 min at room temperature followed by secondary antibody BrightVision Poly‐HRP‐Anti Ms/Rb IgG (both Immunologic; VWR International) for 30 min at room temperature. Staining was developed using Bright‐DAB (Immunologic), and sections were mounted in Pertex mounting medium (Histolab, Askim, Sweden). PSR, VWF, and HIF‐1α slides were digitized with an Olympus dotSlide virtual slide microscope (Olympus, Tokyo, Japan) using a 10× magnification.

Quantification of the digitized stained slices was performed using a custom pipeline in matlab. PSR‐stained slides were converted into the (CIE)Lab color space, with a 3‐axis color system with dimension L for lightness and a and b for the color dimensions, and an absolute threshold was applied to the a‐channel (green to red) to quantify the percentage of collagen‐positive tumor tissue. For all DAB‐stained images (VWF, HIF‐1α), color deconvolution was performed separating the brown DAB staining (Brey *et al.*, 2003). Next, this DAB channel was used to automatically determine a threshold in the tumor ROI using the maximum entropy approach to select positively stained pixels. For the VWF‐stained tissue, the number of positively stained separate elements after an 8‐connected component (bwconncomp, matlab) operation with a minimum size of 50 pixels was counted per mm^2^ to retrieve the vessel density. For HIF‐1α, the amount of positively stained nuclei, separate elements after an 8‐connected component (bwconncomp, matlab) operation with a maximum size of 200 pixels, in the tumor was expressed as a percentage of area.

### 2.5 ROI selections

Tumor ROIs were drawn on the whole‐mount H&E‐stained slides under a microscope by a pathologist (JV) specialized in HPB pathology with 15 years of experience (Fig. 1F) and copied to each separate digitized (Fig. 1G) and quantified (Fig. 1H) staining. For further analysis, average values from this ROI were used to determine percentage of collagen per area (PSR), vessel density per mm^2^ (VWF), and positively stained nuclei as percentage of area (HIF‐1α) for each tumor.

Based on the 3D matching of the pathology specimen to the MRI, the H&E‐based ROIs were projected onto the MRI and propagated onto the DCE and DWI parametric maps for two axial slices (Fig. 1I). Average values from these ROIs were calculated for each quantitative parameter and correlated to the quantified histology. In addition, IHC‐stained and quantified sections could be projected directly onto the MR images along with the quantitative MR parameter maps (Fig. 1J).

Since histopathology matching is not available in clinical routine, separate ROIs were determined solely based on the available imaging. Parametric maps of DCE and DWI were projected on the anatomical mDIXON image using 3d slicer. ROIs were drawn in evidently cancerous pancreas, showing lower perfusion and/or infiltration on the mDIXON image, by a radiologist (MRWE) with 9 years of experience in reading abdominal MR images and a researcher (RK) with 4 years of experience in pancreatic MRI. When necessary, contrast‐enhanced CT scans were viewed next to the MRI imaging for further reference. Care was taken not to include biliary stents in the ROI when present.

### 2.6 Statistical analysis

Statistical analyses were performed in graphpad prism (v5.01; GraphPad Software, La Jolla, CA, USA), r (v3.4.4; R Core Team, 2018, R Foundation for Statistical Computing, Vienna, Austria), and spss (version 24; IBM Corp., Armonk, NY, USA).

Normality of the MRI and histology data was confirmed by the Kolmogorov–Smirnov test (*P* > 0.05). Pearson's correlation coefficients were calculated between IHC and MRI parameters in the pathology ROI and between MRI parameters for the clinical ROI. Values were compared between patients with histology‐derived good‐to‐moderate (grades 1–2) and poor tumor differentiation (grade 3) by Student's *t*‐test. Overall survival (OS) was calculated from the time of the MRI scan to the time of death after discharge or until last follow‐up. Disease‐free survival (DFS) was defined as the time between MRI and progressive disease determined at surgical exploration or return of disease during follow‐up. The maximum difference in log‐rank test approach was used to determine prognostic value of the clinical ROI MRI parameters for OS (Budczies *et al.*, 2012). Kaplan–Meier curves were drawn, and a log‐rank test and Cox proportional hazards model were used to determine significance between groups. A multivariate Cox proportional hazards model was applied for the MRI parameters demonstrating a univariate relation for the patients that underwent resection, adding T‐stage at resection (TNM7), resection margins (R‐stage), patient age, and gender.

## 3 Results

### 3.1 Patients

**Fig. 2:**
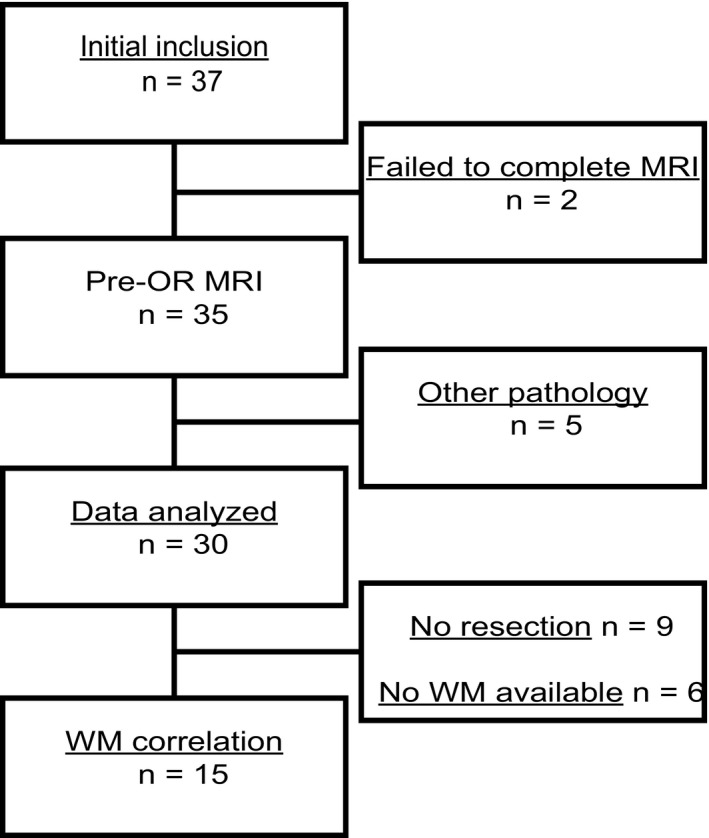
Patient inclusion. Initially, 37 patients were included in the study. Two patients did not undergo MRI scanning, and five patients were excluded after MRI scanning. The resulting data of 30 patients were used for further analyses, of which 21 underwent resection and whole‐mount (WM) histology was available in 15 patients.

From the 37 patients initially included in the study, data of 30 patients could be used for analyses. Two patients did not undergo MRI scanning due to early progression and a late detected contraindication for MRI scanning. Five patients were excluded after MRI scanning, due to different underlying etiologies of the pancreatic lesions determined at histopathological examination of the resection specimen (1 cholangiocarcinoma, 1 nonmalignant intraductal papillary mucinous neoplasm, 1 pancreatitis, and 2 neuro‐endocrine tumors). Of these 30 patients, 21 underwent a resection and whole‐mount histology was available in 15 patients (Fig. 2). Patient demographics are summarized in Table 2.

**Table 2:** Basic patient characteristics for all patients included in the analyses. M1, metastasized disease; LA, locally advanced disease; FU, follow‐up; CI, confidence interval.

Variable	Value (range)
Number of patients	30
Mean age (years)	63 (47–81)
Gender	
Male	18
Female	12
Tumor location	
Head	25
Corpus	2
Tail	3
Tumor diameter PA (mm)	33 (15–55)
Resection	
No	9 [8 M1, 1 LA]
R0	10
R1	11
Differentiation grade	
Well	2
Moderate	8
Poor	9
No resection	9
Missing	2
Time between MRI and Surgery (days)	9 (1–32)
Median FU (months, 95% CI)	41 (36–46)
Median OS (months, 95% CI)	18 (14–22)
Median DFS (months, 95% CI)	8 (1–14)

### 3.2 Quantitative MRI correlates with histology

For the 15 patients from whom whole‐mount histology was available, the whole‐mount H&E‐based tumor ROIs were propagated to the MRI. Resulting median MRI ROI volumes were 2.9 cm^3^ (range 1.5–4.9 cm^3^) for DWI (*n* = 15) and 1.9 cm^3^ (range 1.2–4.9 cm^3^) for DCE and R2* (*n* = 15). Since the ROI was propagated to two MRI slices and the slice thickness was different between the MRI sequences for DCE/T2* and DWI, the resulting ROI volumes were different. For DCE analysis, a median of 89% (range 57–100%) of the voxels showed a reliable fit result (*v*_e_ < 1.0) in the pathology ROI. IVIM fits resulted in a median *R* ^2^ in the pathology ROI of 0.73 (range 0.34–0.89).

**Fig. 3:**
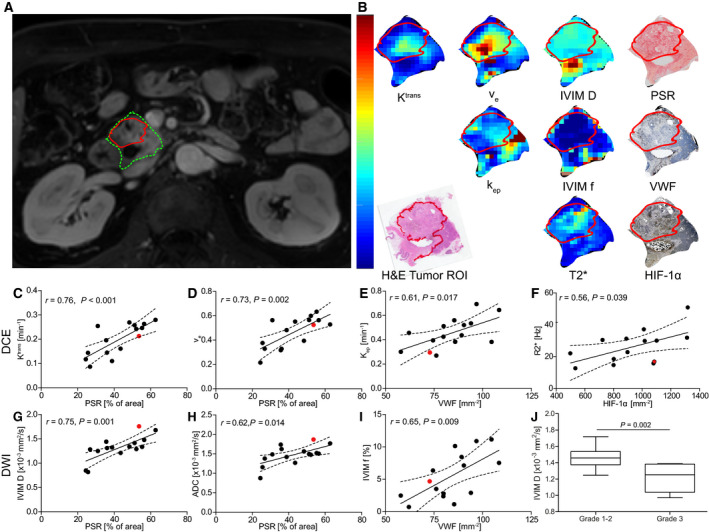
Correlations between histology and quantitative MRI‐derived parameters. (A) Anatomical MRI with pancreatic head (green) and tumor ROI (red). (B) Quantitative MRI parameter maps and histology depicted for one patient. Correlation plots for the DCE parameters (C–E), R2* (F), and DWI parameters (G–I) that demonstrated a significant correlation with histology (Pearson's correlation coefficient *r* < 0.05, *n* = 15). The patient from A and B is depicted in red. (J) Tumors with histological differentiation grades 1–2 (*n* = 8) demonstrated significantly higher diffusivity (IVIM *D* 1.47 ± 0.17 × 10^−3^ mm^2^·s^−1^ vs. 1.15 ± 0.22 × 10^−3^ mm^2^·s^−^, *P* = 0.002, Student's *t*‐test), compared to grade 3 tumors (*n* = 7). Error bars showing min‐max.

We then set out to assess whether the three relevant biological characteristics of PDAC – collagen fraction, vessel density, and hypoxia – could be assessed with functional MR. Mean parameter values for DCE, R2*, and DWI and relevant correlation coefficients with parameters derived from histology are summarized in Table 3. An example of a patient MRI showing the quantitative parameter maps with corresponding histopathology is shown in Fig. 3A,B. We observed a significant correlation between PSR, as a measure of collagen fraction, and DCE *K* ^trans^ and *v*_e_ (Fig. 3C,D) as well as IVIM *D* and ADC (Fig. 3G,H). VWF, quantifying vessel density, correlated significantly with DCE *k*_ep_ (Fig. 3E) and IVIM *f* (Fig. 3I). The amount of HIF‐1α positively stained nuclei, as a measure of hypoxia, demonstrated a significant association with R2* (Fig. 3F). There was a significant difference in IVIM *D* between tumor differentiation grades (Fig. 3J).

**Table 3:** Mean values and correlations between MRI and histology‐derived parameters in the pathology ROI. *r*, Pearson's correlation coefficient; *v*_p_, blood plasma volume.

Parameter		PSR Collagen fraction (%)		VWF Vessel density (mm^−2^)		HIF‐1α Hypoxia (mm^−2^)	
	Mean ± SD	42.81 ± 12.50		83.86 ± 14.21		933.5 ± 249.3	
		*r*	*P*	*r*	*P*	*r*	*P*
*K* ^trans^ (min^−1^)	0.20 ± 0.07	**0.76**	**< 0.001**	0.10	0.717	0.38	0.184
*k*_ep_ (min^−1^)	0.45 ± 0.12	0.10	0.722	**0.61**	**0.017**	0.09	0.768
*v*_e_ (–)	0.46 ± 0.13	**0.73**	**0.002**	−0.44	0.098	0.28	0.329
*v*_p_ (–)	0.03 ± 0.02	0.53	**0.042**	0.17	0.551	0.26	0.360
R2* (Hz)	28.56 ± 14.31	0.19	0.510	0.05	0.850	**0.56**	**0.039**
ADC (10^−3^ mm^2^·s^−1^)	1.48 ± 0.25	**0.62**	**0.014**	0.16	0.568	0.33	0.254
IVIM *D* (10^−3^ mm^2^·s^−1^)	1.32 ± 0.25	**0.75**	**0.001**	−0.15	0.598	0.35	0.223
IVIM *f* (%)	5.49 ± 3.57	−0.23	0.416	**0.65**	**0.009**	−0.01	0.979
IVIM D* (10^−3^ mm^2^·s^−1^)	94.02 ± 55.51	0.21	0.456	−0.20	0.486	0.17	0.551

Bold values indicates *P* < 0.05

## 4 Quantitative MRI parameters show prognostic potential

For survival analysis, the clinical MRI ROIs from all 30 included patients were used. These ROIs resulted in median surface area of 3.2 cm^2^ (range: 1.8–6.6 cm^2^). For DCE analysis, a median of 85% (range 21–100%) of the voxels showed a reliable fit result (*v*_e_ < 1.0) in the clinical MRI ROI. IVIM fits resulted in a median *R* ^2^ in the clinical MRI ROI of 0.75 (range 0.47–0.92). Correlations between the different MRI parameters are summarized in Table 4. DCE *k*_ep_ and IVIM *f* (*r* = 0.54, *P* = 0.002) demonstrated a positive correlation, and both correlated to vessel density in the comparison to histology.

**Table 4:** Mean MRI parameter values and correlations in the clinical ROI. *r*, Pearson's correlation coefficient, with in bold all values with p<0.05; *v*_p_, blood plasma volume.

Parameter	Mean	SD	*K* ^trans^		*k*_ep_		*v*_e_		*v*_p_		R2*		ADC		*D*		*f*	
			*r*	*P*	*r*	*P*	*r*	*P*	*r*	*P*	*r*	*P*	*r*	*P*	*r*	*P*	*r*	*P*
*K* ^trans^ (min^−1^)	0.22	0.09																
*k*_ep_ (min^−1^)	0.43	0.14	**0.49**	**0.006**														
*v*_e_ (–)	0.53	0.15	**0.71**	**0.000**	−0.20	0.290												
*v*_p_ (–)	0.03	0.02	**0.61**	**0.000**	**0.42**	**0.023**	0.35	0.060										
R2* (Hz)	28.66	14.13	0.26	0.356	0.05	0.869	0.23	0.410	−0.10	0.722								
ADC (10^−3^ mm^2^·s^−1^)	1.55	0.22	0.03	0.886	0.20	0.299	−0.14	0.454	0.06	0.758	0.13	0.657						
*D* (10^−3^ mm^2^·s^−1^)	1.36	0.18	−0.18	0.344	−0.06	0.765	−0.15	0.425	−0.07	0.706	0.01	0.972	**0.80**	**0.000**				
*f* (%)	5.55	3.26	0.30	0.112	**0.54**	**0.002**	−0.16	0.405	0.16	0.409	0.21	0.462	0.34	0.069	−0.14	0.462		
D* (10^−3^ mm^2^·s^−1^)	80.25	44.39	0.05	0.788	−0.05	0.785	−0.01	0.971	−0.21	0.261	−0.03	0.923	−0.21	0.259	−0.18	0.346	0.32	0.080

Bold values indicates *P* < 0.05.

**Fig. 4:**
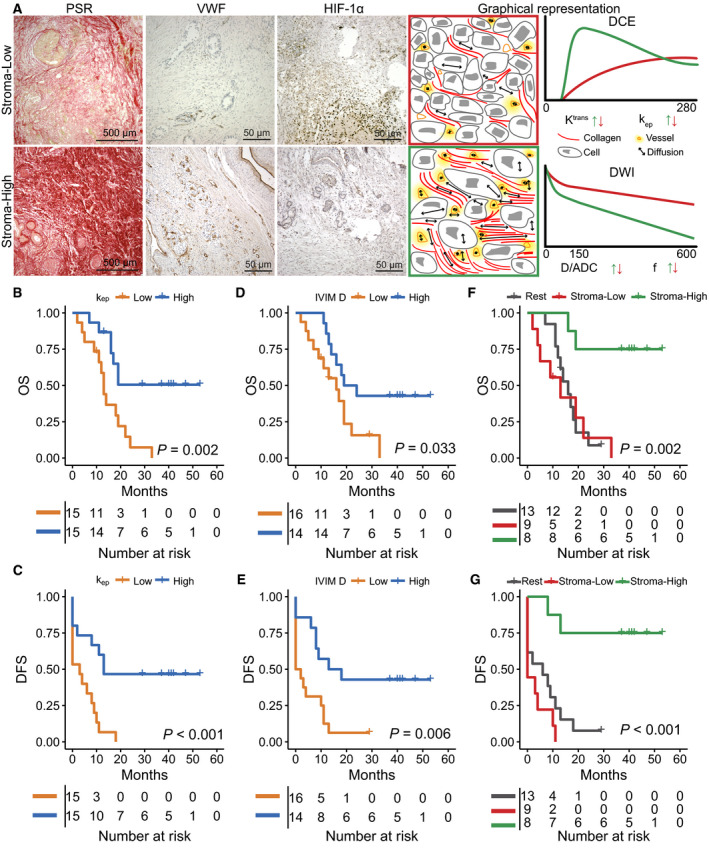
Survival analyses for the entire patient population. (A) The differences between tumor phenotypes (stroma‐high, stroma‐low) are illustrated for two patients for PSR, VWF, and HIF‐1α along with the theoretical signal curves from both DCE and DWI. The stroma‐low phenotype demonstrates low collagen fraction and low vessel density, resulting in DCE to detect reduced contrast transfer to the interstitial space (*K* ^trans^) with low perfusion (*k*_ep_). IVIM demonstrates a lower vessel fraction and a reduction in diffusivity due to the reduced interstitial space. In the stroma‐high phenotype, the increased vessel density and the increase in interstitial space induced by excessive collagen deposition result in higher *k*_ep_ and an increase in both *v*_e_ and *K* ^trans^. IVIM demonstrates a higher vessel fraction and high diffusivity. (B, C) Based on the maximum difference in log‐rank test approach, *k*_ep_ was prognostic for OS (X vs. 13 months, *P* = 0.002, HR: 3.7, *P* = 0.005, *n* = 30) and DFS (13 vs. 3 months, *P* < 0.001, HR: 3.8, *P* = 0.004, *n* = 30), with X being the median survival not yet reached. (D, E) IVIM *D* was prognostic for OS (19 vs. 16 months, *P* = 0.033, HR: 2.5, *P* = 0.043, *n* = 30) and DFS (13 vs. 0 months, *P* = 0.006, HR: 3.0, *P* = 0.016, *n* = 30). (F, G) The combination of *k*_ep_ and IVIM *D* into tumor phenotypes (stroma‐low, stroma‐high) improved the prognostic value for both OS (X vs. 14 months, *P* = 0.002, HR: 9.1, *P* = 0.004, *n* = 30) and DFS (X vs 2 months, *P* < 0.001, HR: 9.3, *P* = 0.003, *n* = 30). With *P*‐values for survival differences being derived from log‐rank tests and for HR from Cox regression.

Based on the maximum difference in log‐rank test approach, we were able to identify prognostic cutoff values for *k*_ep_ and IVIM *D*. Patients with *k*_ep_ > 0.397 min^−1^ (*n* = 15) demonstrated longer OS and DFS compared to patients with lower *k*_ep_ (Fig. 4B,C). The cutoff for IVIM *D* (1.375 × 10^−3^ mm^2^·s^−1^) divided the group into 16 patients with high and 14 with low IVIM *D*, demonstrating longer OS and DFS for the patient with higher tumor diffusivity (Fig. 4D,E).

Combining the findings from the histological correlation and survival analysis, two main phenotypes could be distinguished, a stroma‐high phenotype demonstrating high vessel density and high collagen fraction and a stroma‐low phenotype demonstrating low vessel density and low collagen fraction. In Fig. 4A, the typical difference in tumor biology between these two phenotypes is illustrated. Patients with the stroma‐high phenotype (high *k*_ep_ and high IVIM *D*, *n* = 8) showed longer OS compared to the other patients (Fig. 4F,G).

**Fig. 5:**
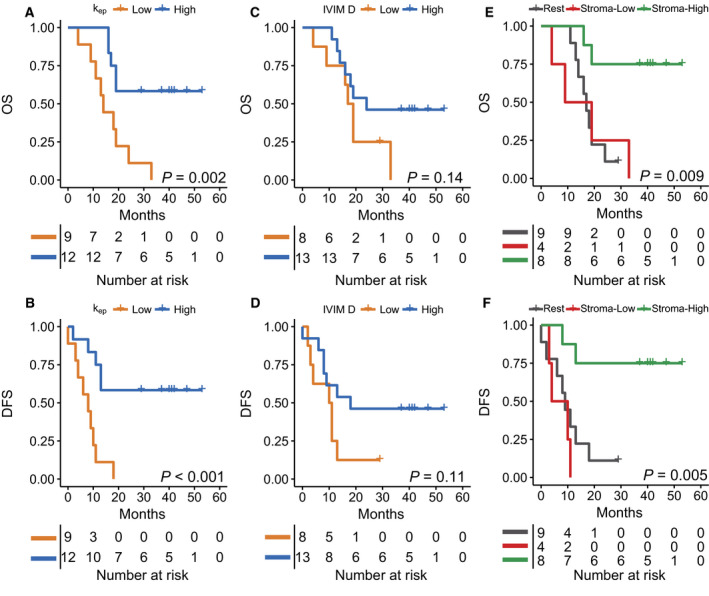
Survival analyses for the patient that underwent resection of the primary tumor. (A, B) *k*_ep_ was prognostic for OS (X vs. 14 months, *P* = 0.002, HR: 4.7, *P* = 0.007, *n* = 21) and DFS (X vs. 8 months, *P* < 0.001, HR: 5.7, *P* = 0.003, *n* = 21). (C, D) IVIM *D* was not prognostic for OS (24 vs 17 months, *P* = 0.14, HR: 2.1, *P* = 0.16, *n* = 21) or DFS (18 vs. 10 months, *P* = 0.11, HR: 2.3, *P* = 0.13, *n* = 21). (E, F) The combination of *k*_ep_ and IVIM *D* into phenotypes was prognostic for both OS (X vs. 17, *P* = 0.009, HR: 7.8, *P* = 0.009, *n* = 21) and DFS (X vs. 9, *P* = 0.005, HR: 7.5, *P* = 0.009, *n* = 21). With *P*‐values for survival differences being derived from log‐rank tests and for HR from Cox regression.

At the time of surgical exploration, nine patients turned out to have metastatic or irresectable disease. No significant differences in imaging parameter between patients with resectable and unresectable tumors were found. However, since the latter were subsequently treated with palliative rather than curative intent, we repeated the survival analysis for patients who underwent a resection of the primary tumor. In this group, *k*_ep_ was still prognostic for OS and DFS (Fig. 5A,B). Multivariate Cox regression demonstrated that *k*_ep_ was an independent predictor for OS (HR = 5.8, *P* = 0.012, *n* = 19) and DFS (HR = 8.0, *P* = 0.022, *n* = 19) in addition to standard clinical parameters. IVIM *D* was not prognostic for OS or DFS in this subset of patients (Fig. 5C,D). The stroma‐high phenotype still showed longer OS and DFS (Fig. 5E,F). Multivariate analysis on the subtypes also showed the added value of the imaging parameters for predicting OS (HR: 19.5, *P* = 0.039) and DFS (HR: 23.5, *P* = 0.02).

## 5 Discussion

In this preliminary study, we found that quantitative MRI parameters correlate with tumor collagen fraction, vessel density, and hypoxia, which are considered important hallmarks in determining the poor outcome of PDAC. Using quantitative MRI, we identified two PDAC phenotypes, stroma‐high and stroma‐low, which were associated with significant differences in prognosis.

Multiple clinical (Bali *et al.*, 2011; Ma *et al.*, 2016; Xu *et al.*, 2017) and preclinical (Wegner *et al.*, 2016, 2017; Wu *et al.*, 2015) studies have investigated the relations between DCE‐derived quantitative parameters and histological tissue properties in PDAC. However, none of these studies performed an extensive pathology matching procedure as done in this study. We found a positive correlation between *K* ^trans^, *v*_e_, and collagen fraction. Although we did not find a correlation between vascular density and *K* ^trans^ as was found in a preclinical setting (Wegner *et al.*, 2017), we did find that *K* ^trans^ is associated with the amount of collagen deposition in the tumor. In addition, when *K* ^trans^ was divided by the extracellular compartment (*v*_e_), which we and others have associated with collagen deposition in a (pre)clinical setting (Bali *et al.*, 2011; Ma *et al.*, 2016; Wegner *et al.*, 2016; Xu *et al.*, 2017), we found that the resulting *k*_ep_ was able to detect the relatively small differences in vascularity between PDAC tumors. This might suggest that vascular flow and permeability in PDAC are more dependent on the tumor micro‐environmental properties associated with a collagen‐rich microenvironment than the actual amount of vessels that are present.

In some parts of the tumor with very low perfusion, information is hard to extract using a perfusion‐based method as DCE, due to the lack of contrast enhancement in these regions. This is not an issue for IVIM *f*, an IVIM‐based measure for perfusion fraction, which also demonstrated a correlation with tumor vascularity from histology. Thus far, only one other study found a positive correlation between IVIM *f* and vessel density in PDAC (Klauss *et al.*, 2015). However, this study also included highly perfused neuro‐endocrine tumors. Although in our study IVIM *f* did show a correlation with both tumor vascularity and *k*_ep_, it did not associate with survival. The limited reproducibility of IVIM *f*, as we demonstrated previously (Gurney‐Champion *et al.*, 2018), and the more limited image quality of the quantitative maps to determine an image‐based ROI could explain this result.

Studies investigating the correlation between DWI, collagen deposition, and cellular density in PDAC have reported contradictory results. Some studies have demonstrated a positive correlation between collagen deposition and ADC (Heid *et al.*, 2016; Klauss *et al.*, 2013), where others demonstrated lower diffusivity in dense fibrosis (Hecht *et al.*, 2017; Ma *et al.*, 2016; Muraoka *et al.*, 2008; Xu *et al.*, 2017) or no correlation between ADC and stromal content (Xie *et al.*, 2015). However, none of these studies included a histological comparison as large and detailed as our whole‐mount approach. Heid *et al.* (2016) demonstrated recently that ADC correlates inversely with cellular density. This would support our current findings since, for PDAC tumors with high cellular density, collagen fraction is lower and vice versa.

Our survival analysis demonstrated that higher tumor diffusivity is a good prognostic factor. This is in line with earlier studies investigating the prognostic value of ADC in PDAC (Heid *et al.*, 2016; Kurosawa *et al.*, 2015). DCE‐derived *k*_ep_ performed even better as prognostic marker in our study. Although tumor vascularity is a known prognostic factor in PDAC (Hoem *et al.*, 2013), so far only one imaging‐based study demonstrated a difference in survival, based on static contrast enhancement on CT (Fukukura *et al.*, 2014). Patients demonstrating a stroma‐high phenotype had better outcome. From a biology perspective, these tumors are characterized by dense collagen content and good vascularization and are relatively well differentiated. This suggests that collagen and tumor stroma can have a protective, or tumor‐constraining, role in PDAC. This is supported by a recent clinical study, indicating that the presence of stroma restrains the progression of basal‐like tumors and improves survival in patients with stroma‐activated and desmoplastic tumor subtypes, whereas for well‐differentiated tumors, survival is reduced when a stromal signal is present (Puleo *et al.*, 2018). In addition, preclinical studies in genetically engineered mouse models revealed that depletion of the tumor stroma as a treatment strategy for PDAC resulted in more aggressive, dedifferentiated tumors and reduced survival (Özdemir *et al.*, 2014; Rhim *et al.*, 2014). In addition, phase I and II trials using IPI‐926 – a Hedgehog inhibitor depleting the tumor‐associated stroma – were stopped early due to detrimental effects: PDAC patients receiving this regimen showed shorter survival (Catenacci *et al.*, 2013). However, whether the differentiation grade of tumor cells defines the stromal content or the stromal cells define the differentiation grade of tumor cells remains to be elucidated. Especially, stroma‐low tumors, where vessel density is low, would also be prone to develop hypoxia, another known prognostic factor in PDAC (Kitada *et al.*, 2003). We could not find a direct correlation between vascularization, diffusivity, and HIF‐1α positivity in our study. R2* on the other hand did show an association with tumor hypoxia, which implies that PDAC hypoxia is driven by a complex combination of factors and could benefit from more targeted imaging strategies (Klaassen *et al.*, 2015).

Some limitations of our study should be taken into account. First, for both histological correlations and survival analysis the number of patients investigated is limited. However, our approach of directly matching the histology to the MR does add to the validity of the found correlations between histology and quantitative imaging. Furthermore, the prognostic value found for DWI is in line with previous findings. Second, the larger voxel size of MRI compared to histology makes comparison of intratumoral regions more difficult, and in our current approach, only one 4‐µm slice was available from histology. We therefore correlated average values derived from only one tumor slice, thereby neglecting intratumor heterogeneity in the current analyses. The addition of a MRI‐based 3D mold to enable more accurate slicing and orientation of the pathology specimen relative to the MRI (Costa *et al.*, 2017) might help to improve the match between MRI and histology and facilitate heterogeneity analysis. Third, the generalizability of our findings could be limited by the variation in acquisition and postprocessing methods available and standardization of these imaging methods is necessary when implementing these techniques on a larger scale (O'Connor *et al.*, 2017; QIBA, 2012, 2019; Taouli *et al.*, 2016).

## 6 Conclusions

In conclusion, quantitative MRI methods are able to quantify tumor collagen fraction, vessel density, and hypoxia in PDAC. Based on the imaging‐derived characteristics, we identified that patients with a stroma‐high phenotypes, described by a high collagen fraction and high vessel density, and demonstrated significantly better outcome compared to other patients. These findings may help to improve stratification of patients for treatment and warrant further research on this topic.

## Conflict of interest

HWML has acted as a consultant for BMS, Eli Lilly and Company, and Nordic Pharma Group/Taiho, and has received unrestricted research grants from Amgen, Bayer Schering Pharma AG, BMS, Celgene, Eli Lilly and Company, GlaxoSmithKline Pharmaceuticals, MSD, Nordic Pharma Group, Philips, and Roche Pharmaceuticals. MFB has received research funding from Celgene. JWW has received research funding from Celgene and Novartis. JS has acted as consultant for Robarts Clinical Trials concerning MRI in Crohn's disease. None of these companies were involved in the design of the study; collection, analysis, or interpretation of the data; drafting of the manuscript; or the decision to submit the manuscript for publication. All other authors declare no conflict of interest.

## Author contributions

RK and AS collected, analyzed, and interpreted the data; wrote the draft manuscript; and prepared the figures. OJG‐C, AJN, and RK optimized the imaging protocols and performed image processing. MRWE and RK reviewed the imaging data. JV, MJV, OJB, GKJH, and AS facilitated and performed the immunohistochemistry and pathology analyses. RK and AS performed immunohistochemistry quantification. GT, MGB, ORB, JWW, CHJE, and MS were clinical investigators and contributed to data collection and patient inclusion. HWML, MFB, JS, and AJN designed and coordinated the study and reviewed the data. All authors contributed to the final manuscript.
